# Human *RSPO1*/R-spondin1 Is Expressed during Early Ovary Development and Augments β-Catenin Signaling

**DOI:** 10.1371/journal.pone.0016366

**Published:** 2011-01-28

**Authors:** Sara Tomaselli, Francesca Megiorni, Lin Lin, Maria Cristina Mazzilli, Dianne Gerrelli, Silvia Majore, Paola Grammatico, John C. Achermann

**Affiliations:** 1 Medical Genetics, Molecular Medicine Department, S. Camillo-Forlanini Hospital, Sapienza–University of Rome, Rome, Italy; 2 Developmental Endocrinology Research Group, UCL Institute of Child Health, London, United Kingdom; 3 Experimental Medicine Department, Sapienza–University of Rome, Rome, Italy; 4 Neural Development Unit, UCL Institute of Child Health, London, United Kingdom; The National Institute of Diabetes and Digestive and Kidney Diseases, United States of America

## Abstract

Human testis development starts from around 42 days post conception with a transient wave of SRY expression followed by up-regulation of testis specific genes and a distinct set of morphological, paracrine and endocrine events. Although anatomical changes in the ovary are less marked, a distinct sub-set of ovary specific genes are also expressed during this time. The furin-domain containing peptide R-spondin1 (RSPO1) has recently emerged as an important regulator of ovary development through up-regulation of the WNT/β-catenin pathway to oppose testis formation. Here, we show that RSPO1 is upregulated in the ovary but not in the testis during critical early stages of gonad development in humans (between 6–9 weeks post conception), whereas the expression of the related genes *WNT4* and *CTNNB1* (encoding β catenin) is not significantly different between these tissues. Furthermore, reduced R-spondin1 function in the ovotestis of an individual (46,XX) with a *RSPO1* mutation leads to reduced β-catenin protein and *WNT4* mRNA levels, consistent with down regulation of ovarian pathways. Transfection of wild-type *RSPO1* cDNA resulted in weak dose-dependent activation of a β-catenin responsive TOPFLASH reporter (1.8 fold maximum), whereas co-transfection of *CTNNB1* (encoding β-catenin) with *RSPO1* resulted in dose-dependent synergistic augmentation of this reporter (approximately 10 fold). Furthermore, R-spondin1 showed strong nuclear localization in several different cell lines. Taken together, these data show that R-spondin1 is upregulated during critical stages of early human ovary development and may function as a tissue-specific amplifier of β-catenin signaling to oppose testis determination.

## Introduction

Sex development is a complex process that requires the integrated interaction of a network of different endocrine and paracrine signaling pathways, transcription factors and steroid hormones, during a critical time period in embryogenesis. In humans, this process starts from around 6 weeks post-conception with the expression of *SRY* in the developing testis and several other critical genes downstream of *SRY* have now been discovered from human and mouse studies [1]–[3].

For many years, testis development was believed to be the more active process. Although the morphological changes in the developing testis and in surrounding structures are more marked than in the developing ovary [4], recent gene expression data from mice have shown that a significant subset of ovary-specific genes are also expressed during this critical stage of embryogenesis [5], [6]. Whilst some of these genes may represent ovary-determining or maintenance genes, data from mice and humans suggest that certain key components of the Wnt signaling pathway, and other potential nuclear targets (e.g. *NR0B1*) may antagonize or oppose testis development [7]–[12]. These effects may be mediated by β-catenin signaling, which has recently been shown to be necessary in the ovary for preventing testis-like characteristics, but to be dispensable in the embryonic testis [13]. Furthermore, Foxl2 seems necessary to maintain the ovary in a differentiated state [14]. Thus, ovary development is likely to be a more active process than originally thought, and disruption of genes in this cascade may lead to complete or partial transdifferentiation of ovarian tissue into testis (e.g. testicular DSD (Disorder of Sex Development) [“XX males”], ovotesticular DSD [“46,XX true hermaphrodites”], respectively).

One of the most significant advances in understanding ovary development came through the discovery of single-gene defects in *RSPO1* (encoding R-spondin1) in several patients with testicular DSD (“SRY negative XX males”), palmoplantar hyperkeratosis and a predisposition to skin tumors (OMIM *609595) [15]. R-spondin1 belongs to a family of secreted furin-like domain containing proteins that are believed to interact with canonical Wnt signaling pathways by influencing externalization of the LRP6 cell membrane receptor, as well as through stabilization of intracellular β-catenin [16]–[18]. R-spondin1 has been shown to be expressed in the mouse ovary at a critical time in development as well as in the developing dermis, kidney and dermal papilla of adult skin [15]. Targeted deletion of *Rspo1* in mice results in testis development in chromosomal XX females [19], [20]. The report of a homozygous splice mutation in *RSPO1* predicted to result in an in-frame deletion of the first furin-like domain in R-spondin1 (p.Ile32_Ile95del) in a patient with 46,XX ovotesticular DSD (formerly “true hermaphroditism”) has provided further evidence for the critical role of this protein in humans [21].

Despite these advances, relatively little is known about the expression of R-spondin1 in early human ovary development or its downstream interactions with components of the Wnt signaling pathway. Here, we show upregulation of R-spondin1 in the human ovary at a critical time in development, augmentation of β-catenin signaling by RSPO1 in a human cell system, and nuclear localization of R-spondin1.

## Results

### RSPO1 expression increases at key stages of early human ovary development

**Figure 1:**
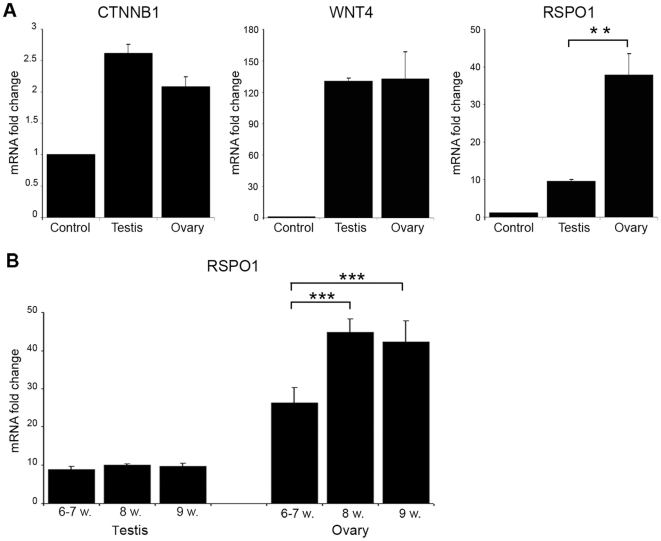
*RSPO1* expression increases at key stages of early human ovary development. (A) Analysis of *CTNNB1*, *WNT4*, and *RSPO1* in human fetal gonads between 6–9 weeks post-conception. Testis and ovary samples showed higher expression of *CTNNB1* (ANOVA, p<0.001), *WNT*4 (ANOVA, p<0.01) and *RSPO1* (ANOVA, p<0.001) compared to control. Significantly higher expression of *RSPO1* was detected in the ovary compared to testis (**p<0.01). (B) Analysis of *RSPO1* expression levels in the testis and ovary during this period of development. A significant increase in *RSPO1* was found in the ovary across this time course (ANOVA, P<0.0001; 8w >6–7w and 9w >6–7w, both ***p<0.001) (control, 8 wpc heart).

Analysis of *CTNNB1* (ENSG00000168036), *WNT4* (ENSG00000162552), and *RSPO1* (ENSG00000169218) in human fetal gonads between 6–9 weeks post-conception showed significantly higher expression of *RSPO1* in the developing ovary compared to testis, whereas no significant differences in the expression of *CTNNB1* or *WNT4* were seen (Fig. 1A). Analysis of *RSPO1* expression levels across this time course revealed an incremental rise in mRNA levels in the ovary from 6-7w to 8w (Fig. 1B). In contrast, *RSPO1* mRNA levels were lower in the developing testis and did not change throughout these key developmental stages (Fig. 1B).

### β-catenin and *WNT4* expression in RSPO1 altered ovotestis

**Figure 2:**
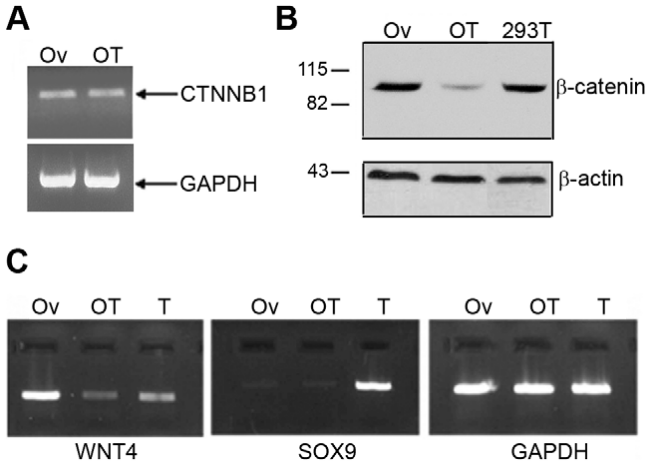
Expression analysis of factors crucial in gonadal development following disruption of RSPO1. (A) *CTNNB1* mRNA levels, determined by RT-PCR and compared to *GAPDH* mRNA levels, seem to have no difference between normal ovary (Ov) and patient's ovotestis (OT). (B) β-catenin protein expression appeared decreased in ovotestis tissue compared to control ovary and HEK293T cell line, in an immunoblot assay (as shown in Tomaselli et al, 2008 [21]). The weak residual band may represent extracellular β-catenin, which is implicated in cell-cell adhesion. (C) *WNT4* and *SOX9* mRNA levels were tested by RT-PCR and compared to *GAPDH* transcript levels. *WNT4* was dramatically reduced in the patient's ovotestis (OT) in comparison to control ovary (Ov). SOX9 signal was detected in control testis (T), but did not appear upregulated in the ovotestis (OT) sample compared to the normal ovary (Ov).

Mutant (MT) R-spondin1 lacks the first furin-like domain and has been reported to be responsible for abnormal ovary development in a patient with 46,XX ovotesticular DSD [21]. The effects of wild-type (WT) and mutant (MT) R-spondin1 on the expression of the *CTNNB1* gene and its encoded protein, β-catenin, were studied *in vivo* using RNA and protein obtained from the patient's ovotestis (OT) and from normal ovary (Ov) control. RT-PCR analysis showed no noticeable difference in β-catenin mRNA expression between these two tissues (Fig. 2A). However, the patient's ovotestis showed reduced β-catenin protein expression as compared to a normal ovary and HEK293T cell line used as controls, consistent with a proposed role for R-spondin1 in β-catenin stabilization and previous findings [21] (Fig. 2B). Similar results were seen following transfection of WT or MT RSPO1 expression vectors into HEK293T cells (data not shown). The expression of *SOX9* and *WNT4* genes was also examined *in vivo* by RT-PCR analysis in RNA derived from the patient's ovotestis (OT) and normal ovary (Ov) and testis (T) controls (Fig. 2C). *WNT4* mRNA expression levels were markedly lower in the patient's ovotestis compared to ovary control. *SOX9* mRNA (ENSG00000125398) expression was barely detectable both in wild-type ovary and in ovotestis, but strongly expressed in the testis control (Fig. 2C).

### R-spondin1 augments β-catenin signaling in human cell lines

**Figure 3:**
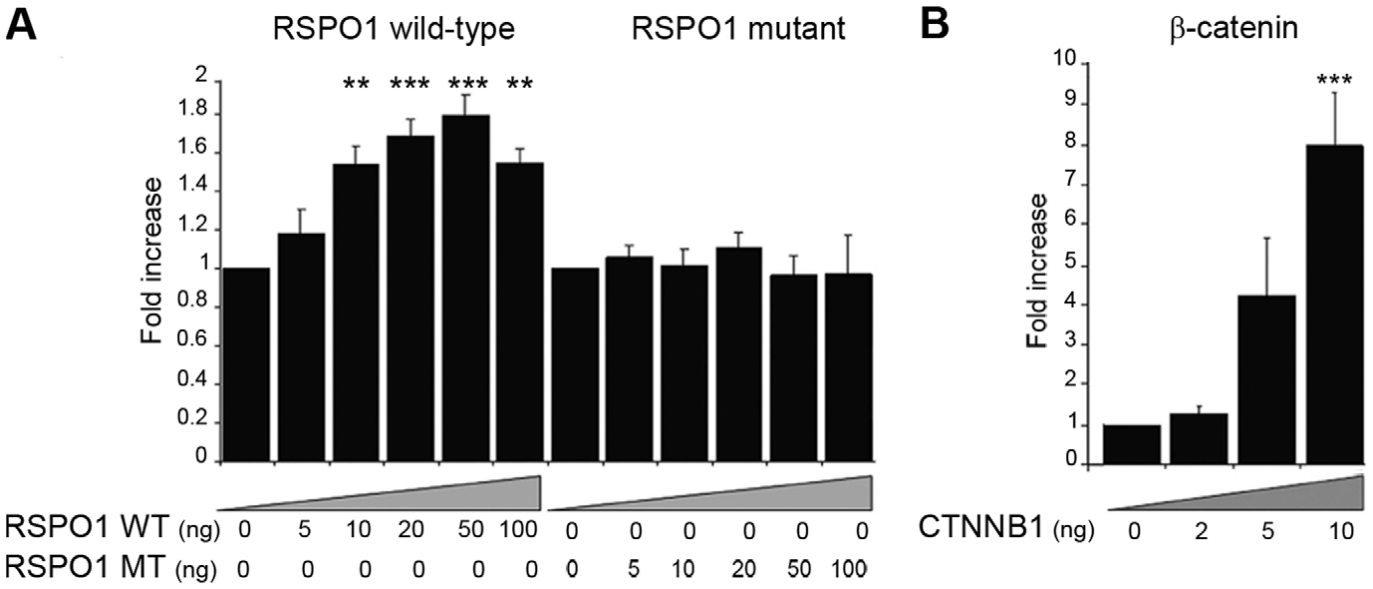
R-spondin1 weakly activates a β-catenin-responsive promoter. (A) The effect of WT RSPO1 on TCF-dependent transcriptional activation (left panel) was compared with that of a naturally-occurring RSPO1 mutant (MT) vector generating a protein lacking the first furin domain (right panel), using embryonic kidney tsa201 cells and the TOPFLASH reporter construct. Transient gene expression assays of increasing concentrations of plasmids encoding wild-type (WT) or mutant (MT) RSPO1 (0–100 ng/well) showed a mild activation (maximum 1.8-fold) of the TOPFLASH reporter with WT *RSPO1* vector (ANOVA, p<0.001) and a complete lack of activity for the mutant. (B) Dose-dependent activation of the TOPFLASH reporter with increasing doses of beta-catenin (0–10 ng/well) (ANOVA, p<0.001). Luciferase data are reported as a mean ± SEM of at least three triplicate experiments, standardized for Renilla co-expression (compared to basal value, **p<0.01; ***p<0.001).

The ability of wild-type (WT) and mutant (MT, p.Ile32_Ile95del) R-spondin1 to activate Wnt/β-catenin dependent targets was investigated using the TOPFLASH (TCF) reporter system. Transient gene expression assays of increasing concentrations of plasmids encoding WT or MT *RSPO1* showed a modest but significant activation of the TOPFLASH reporter vector for the WT (maximal 1.8-fold) and a complete lack of activity for the p.Ile32_Ile95del mutant plasmid (Fig. 3A). However, this WT activation was considerably lower than that seen when increasing doses of a *CTNNB1 (*β-catenin) expression vector were studied (maximal 7.8 fold) (Fig. 3B).

**Figure 4:**
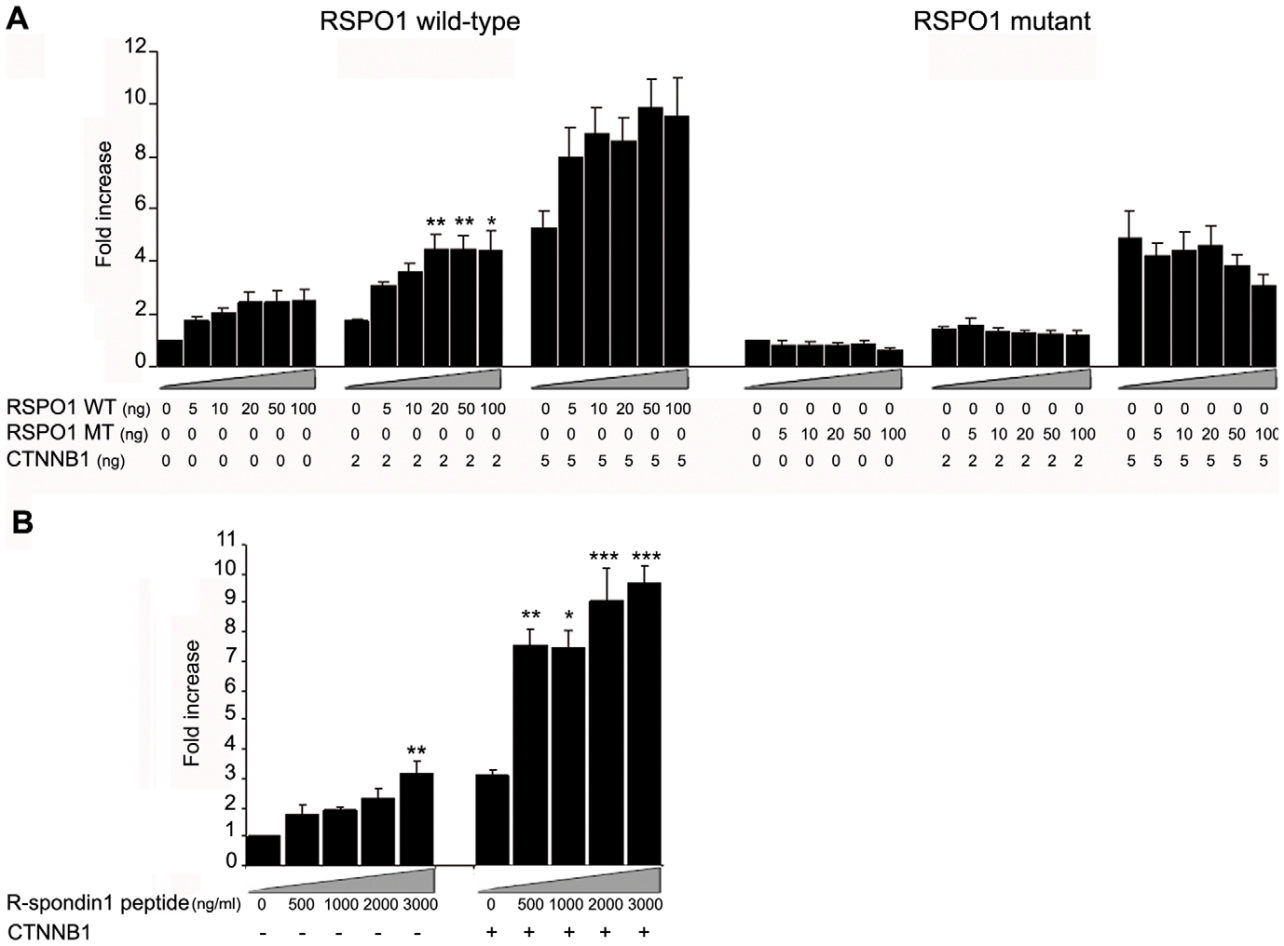
R-spondin1 augments β-catenin signaling. (A) Co-transfection of increasing doses of wild-type (WT) RSPO1 (0–100 ng/well) with β-catenin (0–5 ng/well) showed dose-dependent augmentation of β-catenin signaling (left panel) (ANOVA: CTNNB1 0 ng, p<0.05; CTNNB1 2 ng, p<0.01; CTNNB1 5 ng, p = 0.07). A statistically significant synergistic effect was seen when doses of 10 ng RSPO1 and 50 ng RSPO1 were transfected on the background of increasing doses of CTNNB1 (ANOVA: RSPO1 10 ng, p<0.05; RSPO1 50 ng, p<0.05). No activity was seen after co-transfection of mutant (MT) RSPO1 (0–100 ng/well) (right panel). (B) Relative luciferase activity after stimulation of β-catenin transfected cells with Rspo1 peptide (0–3000 ng/ml) (ANOVA: CTNNB1 -, p<0.01; CTNNB1 +, p<0.001). Luciferase data are reported as a mean ± SEM of at least three triplicate experiments, standardized for Renilla co-expression (*p<0.05; **p<0.01; ***p<0.001 compared to basal value without RSPO1 vector or peptide for that study).

To test whether R-spondin1 could act together with β-catenin, tsa201 cells were co-transfected with a constant concentration of a *CTNNB1* vector expressing β-catenin and with increasing concentrations of WT or MT *RSPO1* (Fig. 4A). Transfection with 2ng or 5 ng of *CTNNB1/*β-catenin plasmid alone activated the TCF reporter by 2-fold and 5.3-fold, respectively (Fig. 4A, left panel). Cells co-transfected with *CTNNB1/*β-catenin and WT RSPO1 vectors showed increased activation of the reporter, up to 10-fold, indicating that R-spondin1 can augment β-catenin activity in a dose-dependent and mildly synergistic manner (Fig. 4A, left panel). This activation was not achieved using a *CTNNB1/*β-catenin plasmid together with MT RSPO1 construct, confirming that R-spondin1 with deletion of the furin1 domain is inactive (Fig. 4A, right panel). Stimulation of *CTNNB1/*β-catenin transfected cells with mouse recombinant R-spondin1 peptide confirmed the ability of R-spondin1 protein to augment β-catenin signaling (Fig. 4B).

### R-spondin1 augmentation of β-catenin signaling is partially inhibited by Dkk1

**Figure 5:**
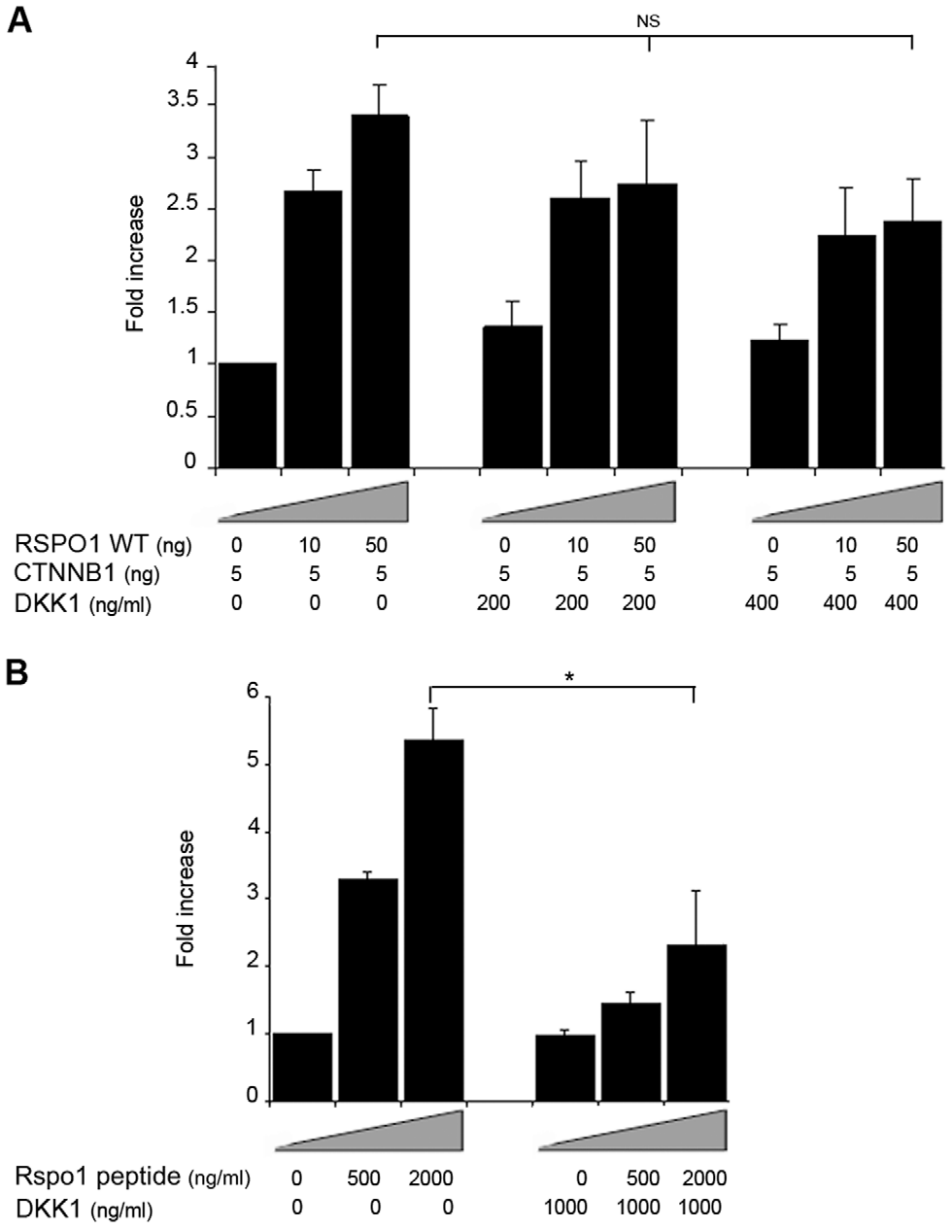
Effect of DKK1 treatment on R-spondin1 augmentation of β-catenin signaling. (A) RSPO1/β-catenin co-transfected cells were treated 2 hours before transfection with different doses of DKK1 (0–400 ng/ml). After 24 h, cells were lysed and assayed for luciferase activity. No significant reduction in stimulation was seen (ANOVA: RSPO1 50 ng, p = 0.15). (B) Cells were treated with DKK1 (0–1000 ng/ml) and stimulated with Rspo1 peptide (0–2000 ng/ml). Luciferase activity was measured 24 h later. Luciferase data are reported as a mean ± SEM of at least three triplicate experiments, standardized for Renilla co-expression. (N.S., not significant; *p<0.05).

In order to investigate further the mechanism of interaction between RSPO1 and β-catenin, cells transfected with RSPO1/R-spondin1 and *CTNNB1/*β-catenin expression vectors were exposed to increasing concentrations of Dickkopf-1 (DKK1) (ENSG00000107984), a potent inhibitor of the Wnt signaling pathway co-receptor LRP6 [17]. DKK1, at concentrations of 0–200–400 ng/ml, showed a non-significant inhibition of RSPO1-augmented β-catenin activation (Fig. 5A). The inhibition was not complete even when higher concentrations of DKK1 were used in the same assay system (data not shown). Exposure to exogenous R-spondin1 peptide and high concentrations of DKK1 (1000 ng/ml) resulted in a significant inhibition of TCF activation by RSPO1/β-catenin (Fig. 5B).

### R-spondin1 shows nuclear and nucleolar localization *in vitro*

**Figure 6:**
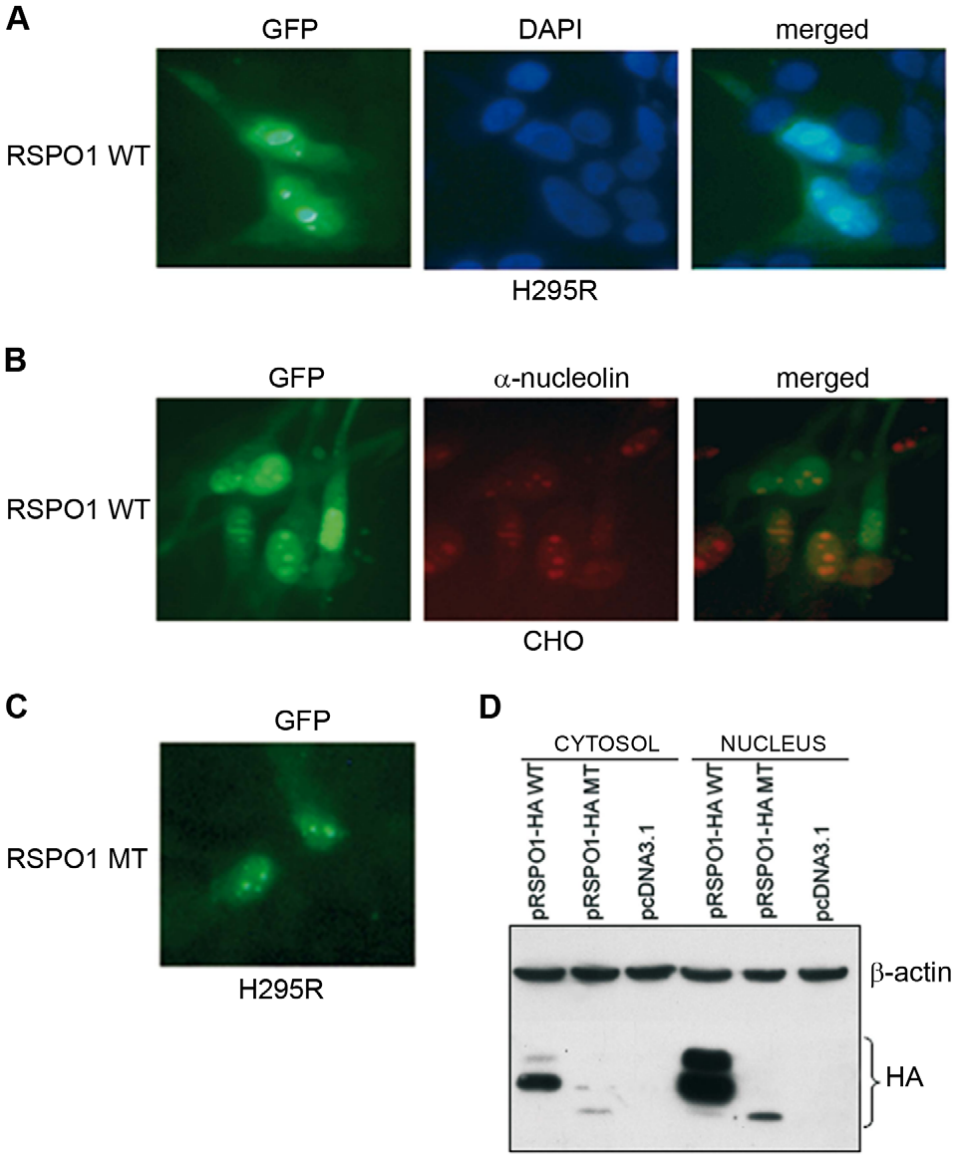
R-spondin1 can show nuclear and nucleolar localization. (A) Immunofluorescent microscopy was used to detect the cellular distribution of a WT pRSPO1-GFP vector in H295R cells (left panel). DAPI stained nuclei and merged images are shown (center and right panels, respectively*)* (magnification 20X). (B) A similar pattern of cellular distribution was obtained in a CHO cell line (left panel). Fluorescent labeling performed with an antibody against C23-nucleolar protein (red) revealed that the GFP-tagged WT RSPO1 protein shows strong nucleolar localization in some of these cells (center and right panels) (magnification 20X). (C) Immunofluorescent analysis of MT pRSPO1-GFP showed similar cellular distribution. (D) The strong nuclear localization was confirmed following Western blot analysis of nuclear and cytosolic extracts prepared from HEK293T cells that had been transfected with either WT or MT pRSPO1-HA constructs.

Plasmids encoding WT or MT RSPO1 (pRSPO1) and expression vectors for different tagged WT and MT proteins (pRSPO1-GFP, pRSPO1-HA) were transiently transfected into several different cell lines (H295R, CHO, HEK293T, NT2/D1). H295R cells expressing GFP-tagged WT RSPO1 showed that the protein was located mostly in the nucleus and partially in the cytoplasm (Fig. 6A). Similar results were seen in other cell lines and using HA-tagged R-spondin1 or an anti-R-spondin1 antibody (data not shown). All transfected cells showed this pattern of distribution. Strong nucleolar staining was confirmed using an α-C23 nucleolin antibody (Fig. 6B). The p.Ile32_Ile95del mutant R-spondin1 showed a similar pattern of cellular localization (Fig. 6C). In order to confirm this result, the subcellular localization of R-spondin1 was also analyzed by Western blot analysis. HEK293T cells were transfected with pRSPO1-HA WT or MT expression vectors and fractionated protein extracts were prepared. The R-spondin1 protein was detected in both the cytosol and nucleus, but a definitely stronger signal was visible in the nuclear fraction (Fig. 6D). The mutant protein also showed stronger nuclear expression, although the overall intensity of bands was less than WT.

## Discussion

The R-spondin family of proteins are a relatively recently identified group of furin-like domain containing factors that are thought to play an important role in tissue development and cellular differentiation. For example, alterations in RSPO4 activity have been reported to cause anonychia in humans [22], whereas disruption of Rspo2 or Rspo3 in mice affects development of several different systems (skeletal, respiratory or placenta defects) [23]. R-spondin1 itself may protect mice from experimental colitis or from chemotherapy or radiotherapy-induced mucosal injury [24]–[26], and it is necessary for normal ductal breast development [27]. However, it has been reports of loss-of-function mutations in R-spondin1 in 46,XX patients with testes (“testicular DSD”) [15] or ovotestes (“ovotesticular DSD”) [21] that have highlighted the action of this R-spondin family member as an important mediator of gonad development. Nevertheless, relatively little is known about the role of R-spondin1 in early human development and the specific mechanisms by which R-spondin1 has its effects.

Studies in the developing mouse gonad have shown that the *Rspo1* gene has a sexually dimorphic expression pattern at specific developmental stages (e12.5–e15.5), with progressively higher expression in the ovary compared to the testis [15], [23], [28]. Analogous data have now been reported in other vertebrates such as chicken, turtle and goat [28], [29]. It is hypothesized that progressive upregulation of *RSPO1* expression in the developing ovary plays an important role in opposing testis development pathways. In the present study, we provide evidence that a similar dimorphic pattern of *RSPO1* gene expression occurs in the human during critical early stages of testis and ovary development (between 6–9 weeks post conception), confirming the likely role of this gene as a key component of the human gonad determination pathway.

The mechanism through which R-spondins have their effects are not fully elucidated but it has been reported that R-spondin proteins act primarily through upregulation of β-catenin and canonical Wnt signaling pathways [30]. The two conserved cysteine-rich furin-like domains of R-spondin proteins are also thought to be important for the stabilization of cytosolic β-catenin [18], [31], [32], which in turn may lead to degradation of Sox9 [33], [34]. Sox9 is an autosomal gene that is upregulated in the developing testis by steroidogenic factor-1 (SF-1) and the principle Y-chromosomal factor, SRY [2]. However, antagonism of Sox9 in the ovary seems necessary to prevent development of testicular tissue, even in the absence of SRY, and now RSPO1, WNT4 and FOXL2 are emerging as important regulators of this process [9], [14]. WNT4 may also act to prevent oocyte apoptosis in the developing ovary [35].

Recent studies in the mouse have provided additional important insight into the specific actions of R-spondin1. For example, deletion of *Rspo1* in XX mice results in the formation of testicular tissue in the ovary (ovotestis) and partial androgenization of female mice, with decreased β-catenin signaling and reduced expression of *Wnt4* [19], [20]. Similarly, deletion of β-catenin in the ovary results in down-regulation of *Wnt4*, but with no effect on *Rspo1* expression. Taken together, these studies suggest that β-catenin is a component between R-spondin1 and Wnt4 [13]. The data from our patient's ovotestis provides direct evidence in humans to support this model, with a reduction in β-catenin protein stability and reduced *WNT4* mRNA in the gonad tissue analyzed, showing in a biological system that these two factors are strikingly related to RSPO1. Further *in vivo* data from patients with *RSPO1* mutations would be needed to confirm this finding, although to date reports of such cases are rare.

Analysis of *SOX9* expression in the ovotestis from our patient did not show any increase in *SOX9* mRNA levels. This finding also supports mouse studies described above, as *Sox9* mRNA was not increased following deletion of either *Rspo1* or *Ctnnb1*/β-catenin in the developing ovary [19], [20], [36]. These data reinforce the hypothesis that R-spondin1 does not regulate transcription of *SOX9* directly, but influences it at the protein level. Unfortunately, it was not possible to undertake measurements of SOX9 protein in our patient sample. Nevertheless, it seems likely that increased SOX9 stability in the ovary following reduction in RSPO1 or β-catenin is a central mechanism leading to testis or ovotestis development within these tissues.

As β-catenin-mediated activation is fairly ubiquitous throughout cellular development and function, the R-spondin family of proteins may play an important role in mediating tissue-specific events. By using human cell-based assay systems, we have shown that R-spondin1 can act to augment β-catenin signaling in a dose-dependent manner. This effect could have a significant biological effect at times when relative dosage effects of sex-determining genes and proteins are important. It is emerging that gene dosage effects are crucial during early gonad development in humans and other species. Furthermore, we have provided the first evidence in humans to shown that expression levels of *CTNNB1* and *WNT4* mRNA are similar in testes and ovaries during these critical stages of early ovary development. Thus, R-spondin1 may act as a tissue (ovary) and time specific amplifier of β-catenin activity. By our functional experiments we have also shown that the mutant RSPO1 protein in our patient was not able to activate the TCF promoter, confirming *in vitro* an essential role of the furin domain for RSPO1 activity or stability.

R-spondin proteins may also interact with members of the Wnt pathway in a synergistic manner. For example, it has been reported that human Rspo1 synergizes with Wnt3a to stabilize β-catenin and that this synergy occurs in the extracellular environment [18], [32], [37]. Generally, R-spondins are believed to be secreted proteins that can modulate Wnt signaling by antagonizing internalization of the Wnt co-receptor, LRP6 [16]. Mechanistically R-spondin1 may compete with Dickkopf-1 (DKK1) for binding to Kremen, which allows LRP6 to become available to upregulate Wnt signaling [17]. To investigate whether the synergistic effect between RSPO1 and β-catenin could be related to R-spondin1-regulated levels of LRP6 on the cell surface, we treated RSPO1/β-catenin transfected cells with different doses of DKK1, a potent inhibitor of LRP6. DKK1 reduced the augmented β-catenin activity induced by RSPO1/R-spondin1, consistent with a significant interaction with LRP6, but did not abolish it completely. These data differ from the complete loss of reporter activity with DKK1 in studies of Wnt3a/RSPO1 synergy [18], and suggest that residual TCF activity could be related to an additional and alternative mechanism of RSPO1 action that is unrelated to inhibition of Kremen/DKK1-dependent LRP6 internalization. R-spondin1 has been shown to contain a carboxyl-terminal putative nuclear localization signal [16], [38] and we have used several different experimental approaches to show strong nuclear and nucleolar localization of R-spondin1 in several different cell-based systems. Whether these direct nuclear effects of R-spondin1 are important at key stages of gonad development is not known, although this could represent an additional mechanism for the effect of this protein in regulating ovary development and in preventing activation of testis-specific pathways. Of note, the p.Ile32_Ile95del mutant R-spondin1 showed a reduced expression level compared to the wild-type protein, by both western blotting and immunofluorescence analysis. This lower expression could be due to reduced stability of the mutant protein lacking the furin domain, and could have contributed in part to its impaired activation of the TCF promoter.

Taken together, these findings confirm that R-spondin1 is a key regulator of ovary development in humans and that this factor may represent a tissue- and time-specific modulator of β-catenin signaling. Our results support the model that ovary-specific genes, such as *RSPO1*, are actively upregulated at critical stages of development to oppose pathways that would otherwise lead to testis development, by several different mechanisms of action.

## Materials and Methods

### Ethics statement

Anonymized human embryonic/fetal material was obtained from the Medical Research Council-Wellcome Trust Human Developmental Biology Resource with written consent and ethical approval from the local Research Ethics Committee of University College London Hospitals and the Royal Free Hospital Research Ethics Committee (08/H0712/34). We obtained tissue samples for genetic and molecular studies after receiving informed consent from the patient, following approval from the Research Ethics Committee of the San Camillo-Forlanini Hospital.

### Fetal gonad RNA preparation

Human testes and ovaries (n = 3 each) from Carnegie stage (CS) 18–21 (6–7 weeks post conception, wpc), Fetal stage 1 (F1) (8wpc) and F2 (9wpc) were obtained on ice and preserved in RNA later (Ambion). Fetal heart tissue (8wpc) was used as a control. RNA was extracted using the Trizol method and the concentration and ratio of absorbance at 260 nm to 280 nm (A260/A280 ratio) were measured using a NanoDrop ND-1000 Spectrophotometer (NanoDrop Technologies, Witec, Littau, Switzerland). First-strand cDNA was synthesized using the SuperScript II Reverse Transcriptase (Invitrogen) and random primers according to the manufacturer's instructions. The amount of input RNA in each reaction was calculated to be 200 ng.

### qRTPCR of *CTNNB1*, *WNT4* and *RSPO1*

Primers and TaqMan® probes were obtained for *CTNNB1*, *WNT4*, and *RSPO1* (Applied Biosystems). Amplification was performed in a total volume of 20 µl per reaction using TaqMan® Gene Expression Master Mix and a StepOne Real-Time PCR system (Applied Biosystems). Thermocycling conditions consisted of an initial step of 2 min at 50°C, denaturation of 10 min at 95°C, followed by 40 cycles of 95°C for 15 s and 60°C for 1 min. Glyceraldehyde-3-phosphate dehydrogenase (*GAPDH*) was used for normalization and relative quantification of gene expression was performed according to the 2^-ΔΔCt^ method [39]. Results are expressed as fold change above control.

### RT-PCR of ovotestis

Total RNA was isolated from patient's gonadal tissue and from normal human ovary and testis using the Chomczynski and Sacchi extraction protocol [40]. RT-PCR was performed (GeneAmp RNA PCR Kit, Applied Biosystems) and *CTNNB1* (encoding β-catenin), *SOX9* and *WNT4* were amplified using gene-specific primers (sequences and conditions available on request). RT-PCR of *GAPDH* was used as a positive control.

### Western blots

Western blots to assess total β-catenin expression were performed using total proteins extracted from patient's gonadal tissue and normal human ovary as described previously, using a mouse monoclonal anti-β-catenin antibody (Santa Cruz Biotechnology, Santa Cruz, CA) [21].

### DNA constructs

A DNA fragment encoding full-length wild-type (WT) R-spondin1 was amplified from normal ovary tissue. A corresponding DNA fragment encoding mutant (MT, p.Ile32_Ile95del) human R-spondin1 was amplified from the ovotestis of a patient with ovotesticular DSD [21]. The amplified regions were verified by sequencing and cloned into a pcDNA3.1(+) plasmid (Invitrogen) for expression in mammalian cells (pRSPO1 WT and MT). These cDNAs were also cloned into a pEGFP-N1 vector (Clontech) to produce mutant fusion proteins with a green fluorescent protein (GFP) tag at the carboxyl-terminus of RSPO1 (pRSPO1-GFP WT and MT), as well as into a pcDNA3.1(+) vector to generate wild-type or mutant RSPO1 with an in-frame fusion to a carboxyl-terminal hemagglutinin (HA) epitope tag (pRSPO1-HA WT and MT). The expression vector encoding wild-type β-catenin was generated from full-length human *CTNNB1* (IMAGE Consortium CloneID 6151332).

### Cell culture

Human embryonic kidney 293T (HEK293T) cells (American Type Culture Collection, ATCC), human embryonic kidney tsa201 cells [41], NT2/D1 human embryonal testicular carcinoma cells (ATCC), and chinese hamster ovary (CHO) cells (ATCC) were routinely maintained in Dulbecco's modified Eagle's medium (DMEM) supplemented with 10% fetal bovine serum (FBS), 100 U/ml penicillin and 100 µg/ml streptomycin in 5% CO_2_ at 37°C (10% CO_2_ for NT2/D1). NCI-H295R human adrenal carcinoma cells (ATCC) were grown in DMEM/F12 1∶1 containing 2.5% Nu-Serum and ITS+ Premix (1X) (BD Biosciences).

### Transient gene expression assays

Transient gene expression assays were performed using Lipofectamine2000 (Invitrogen) and a Dual-Luciferase Reporter Assay System (Promega) in accordance with the manufacturer's instructions. Cells (tsa201) were seeded in 96-well plates (TPP) 24 h prior to transfection. Varying amounts (0–100 ng/well) of either WT or MT pRSPO1 plasmids were transfected with or without varying amounts (0–5 ng/well) of β-catenin vector. The TOPFLASH reporter plasmid (100 ng/well), containing TCF-binding sites linked to luciferase, was used to assess target gene activation [42]. Co-transfection of pRLSV40 *Renilla* luciferase (Promega) was used as an internal control of transfection efficiency. Luciferase assays were performed 24 hours after transfection using a FLUOstar Optima fluorescence microplate reader (BMG Labtech) and luciferase activity was normalized to *Renilla* co-expression. Results are shown as the mean ± SEM for triplicate samples of at least three independent experiments.

### Recombinant R-spondin1

Recombinant mouse R-spondin1 (0–3000 ng/ml final concentration) (R&D Systems) was added to culture media 24 hours prior to luciferase assay and 48 hours prior to immunocytochemistry studies.

### LRP6 inhibition

Human recombinant DKK1 protein (0–1000 ng/ml final concentration) (R&D Systems) was used to block LRP6 activity. This peptide was added to cultured media 2 hours prior to transfection or stimulation.

### Cellular immunohistochemistry

Empty vector, WT and MT pRSPO1, p-RSPO1-GFP, or pRSPO1-HA expression vectors (0.8 µg/chamber) were transfected into several different cell lines (HEK293T, tsa201, NT2/D1, CHO and H295R) seeded in 8-chamber slides using Lipofectamine2000 (Invitrogen). After 24–48 hours, cells were fixed and permeabilized with TritonX-100 (0.3%) prior to immunohistochemistry using standard protocols. The following primary antibodies were used: rabbit polyclonal anti-HA (1∶25) (Sigma), human/mouse monoclonal anti-RSPO1 (1∶150-1∶200) (R&D Systems) and rabbit polyclonal α-C23 anti-nucleolin (1∶100) (Santa Cruz Biotechnology). Nuclear counter staining was performed with Vectashield containing DAPI (Vector Laboratories). Cells were visualized on a Zeiss Axioskop microscope and images captured using a ZeissAxiocam camera.

### Subcellular localization by Western blot analysis

HEK293T cells were transiently transfected with either WT or MT pRSPO1-HA expression vectors (0.8 µg/well) using Lipofectamine 2000 (Invitrogen). After 48–72 hours, cytoplasmic and nuclear protein extracts were prepared using a modified version of the Dignam protocol [43]. Protein concentrations were measured by the BCA assay (Bio-Rad Laboratories). Fifty micrograms of protein extract from each sample was subjected to SDS/PAGE, transferred to polyvinylidene fluoride (PVDF) membranes (Millipore) and probed with the following antibodies: anti-HA probe (1∶500) (sc-7392, Santa Cruz Biotechnology Inc.) or anti-R-spondin1 (1∶300) (AF3474, R&D Systems). Bound antibodies were detected with enhanced chemiluminescence (ECL kit, Amersham). Apparent molecular weight values were estimated using the ColorBurst Electrophoresis Marker (Sigma). β-actin (clone AC-15, Sigma) hybridization was performed as a control for protein loading.

### Statistical analysis

All values are expressed as mean ± SEM unless indicated otherwise. Comparison of means between multiple groups was performed using one-way analysis of variance (ANOVA) with Bonferroni corrections for multiple comparisons where significant differences were found. Comparison of means between two groups was undertaken using unpaired two-tailed Student's t-tests. *P* values were calculated using GraphPad and the statistical analysis function in Microsoft Excel® (2007 version). Significance is expressed at *p≤0.05; **p≤0.01; ***p≤0.001.
